# Analyses of Sensitivity to the Missing-at-Random Assumption Using Multiple Imputation With Delta Adjustment: Application to a Tuberculosis/HIV Prevalence Survey With Incomplete HIV-Status Data

**DOI:** 10.1093/aje/kww107

**Published:** 2017-01-10

**Authors:** Finbarr P Leacy, Sian Floyd, Tom A Yates, Ian R White

**Affiliations:** 1 Division of Population Health Sciences, Royal College of Surgeons in Ireland, Dublin, Ireland; 2 MRC Biostatistics Unit, Cambridge Institute of Public Health, School of Clinical Medicine, University of Cambridge, Cambridge, United Kingdom; 3 MRC Tropical Epidemiology Group, Department of Infectious Disease Epidemiology, Faculty of Epidemiology and Public Health, London School of Hygiene and Tropical Medicine, London, United Kingdom; 4 Centre for Infectious Disease Epidemiology, Research Department of Infection and Population Health, University College London, London, United Kingdom

**Keywords:** causal mediation analysis, incomplete data, nonignorable nonresponse, sensitivity analysis

## Abstract

Multiple imputation with delta adjustment provides a flexible and transparent means to impute univariate missing data under general missing-not-at-random mechanisms. This facilitates the conduct of analyses assessing sensitivity to the missing-at-random (MAR) assumption. We review the delta-adjustment procedure and demonstrate how it can be used to assess sensitivity to departures from MAR, both when estimating the prevalence of a partially observed outcome and when performing parametric causal mediation analyses with a partially observed mediator. We illustrate the approach using data from 34,446 respondents to a tuberculosis and human immunodeficiency virus (HIV) prevalence survey that was conducted as part of the Zambia–South Africa TB and AIDS Reduction Study (2006–2010). In this study, information on partially observed HIV serological values was supplemented by additional information on self-reported HIV status. We present results from 2 types of sensitivity analysis: The first assumed that the degree of departure from MAR was the same for all individuals with missing HIV serological values; the second assumed that the degree of departure from MAR varied according to an individual's self-reported HIV status. Our analyses demonstrate that multiple imputation offers a principled approach by which to incorporate auxiliary information on self-reported HIV status into analyses based on partially observed HIV serological values.

Missing data are common in epidemiologic studies and can lead to substantial bias and misleading inference when inadequately handled. Incomplete data are frequently analyzed only under the missing-at-random (MAR) assumption when they may more plausibly be missing not at random (MNAR). Data are said to be MAR if, conditional on the observed values, missingness of any variable does not depend on the unobserved values (1). Because the MAR assumption cannot be verified from the observed data, it is important to perform sensitivity analyses that assess the impact on the study results of departures from this assumption. However, methods for implementing structured sensitivity analyses are in need of further development and wider dissemination (2). This article reviews the procedure of multiple imputation with delta adjustment and demonstrates how it can be used to assess sensitivity to departures from MAR, both when estimating the prevalence of a partially observed outcome and when performing parametric causal mediation analyses with a partially observed mediator using the approach of Valeri and VanderWeele (3). Mediation analysis allows researchers to explore alternative mechanisms for a given outcome-exposure relationship via third variables and is becoming an increasingly popular tool in epidemiologic research.

We applied the delta-adjustment approach to data from a survey on the prevalence of tuberculosis (TB) and human immunodeficiency virus (HIV) that was conducted as part of the Zambia–South Africa TB and AIDS Reduction (ZAMSTAR) Study (4). We wished to obtain overall and sex-specific estimates of HIV prevalence and investigate the mediating influence of HIV status on the relationship between educational attainment and active pulmonary TB.

Missingness of the HIV test result data is most plausibly MNAR, because prior knowledge or strong beliefs about one's status influence test acceptance. Evidence from several recent longitudinal studies suggests that individuals who have previously tested HIV-positive may be more likely to refuse testing subsequently compared with individuals who were HIV-negative when last tested (5–8). Such individuals may refuse testing because they fear further disclosure of their status to others. Some authors have advocated the collection of additional auxiliary information on prior testing behavior (8) to adjust for this, but there is little guidance on how to incorporate this information into the final analysis; current ad hoc approaches include supplementing the partially observed HIV serological values with self-reported values. By including self-reported HIV status in the imputation model for incomplete HIV serological values we demonstrate a novel and principled approach to incorporating this information that builds on current guidelines from the World Health Organization and the United Nations Programme on HIV and AIDS for handling missingness of HIV status data (9).

Collecting information on past HIV testing behavior, including the self-reported result of the most recent HIV test, also provides an opportunity to conduct more nuanced sensitivity analyses as it is likely that rates of HIV test acceptance differ within groups defined on the basis of self-reported HIV status. To this end, we present results from 2 types of sensitivity analysis: the first assumed that the degree of departure from MAR was the same for all individuals with missing HIV serological values, and the second assumed that the degree of departure from MAR varied according to an individual's self-reported HIV status. To our knowledge, these are the first sensitivity analyses of this type to be reported in the literature.

## METHODS

### ZAMSTAR Study

We used data from a TB/HIV prevalence survey conducted as part of the ZAMSTAR Study (4). This survey aimed to include approximately 4,000 adults aged 18 years or older in each of 16 trial communities in Zambia and 8 communities in the Western Cape province of South Africa. We restricted our analyses to the 34,446 adult participants with an evaluable TB sputum sample among the 16 trial communities in Zambia.

Information on HIV status was available from 2 sources. All survey participants were offered point-of-care, rapid HIV testing as part of the study, yielding a partially observed variable for HIV status based on serological analysis. Participants were also asked about prior HIV tests, yielding a fully observed, self-reported, auxiliary variable with 4 categories: HIV-positive, HIV-negative, refused to disclose the result of the most recent HIV test, and never tested. Data were also collected on a large number of sociodemographic and socioeconomic variables and on prior diagnosis, symptoms, and treatment for TB and/or HIV. Data collected on highest school grade completed were used to create an educational-attainment exposure variable with the following 5 categories: none, primary (less than grade 8), lower secondary (grade 8 or 9), upper secondary (grade 10, 11, or 12), and college/university.

Among participants with an evaluable TB sputum sample, 31.8% had missing HIV serological values. In order to create a data set with univariate missingness, we deleted 648 (1.2%) observations that had missing values on any other variable included in the final imputation model. Omitting these observations had no impact on inference under the MAR assumption (data not shown). Communities were grouped into 4 noncontiguous regions characterized by their annual risk of TB infection (defined by the percentage of schoolchildren with a positive tuberculin test in a 2005 tuberculin skin test survey in all 24 trial communities (10)) and whether they were urban, rural, or located in Lusaka, the capital city.

### Multiple imputation

Multiple imputation involves first specifying a distribution for the unobserved data given the observed data. Multiple complete data sets are produced by taking random draws from this distribution. Each imputed data set is analyzed using standard methods, and point estimates and standard errors for the quantities of interest are aggregated across the imputed data sets using Rubin's rules. Standard implementations assume that the missing data are MAR. A comprehensive treatment of the underlying statistical theory can be found in Rubin (11).

We first describe a standard implementation of multiple imputation under the MAR assumption for a single incomplete variable. We then show how the delta-adjustment procedure extends this approach to allow for multiple imputation under alternative MNAR assumptions by modifying the values imputed under a MAR assumption so that they differ from the observed values in a specified way.

### Multiple imputation under the MAR assumption

#### MAR assumption

Suppose that we have a vector of fully observed variables ***X*** and a single partially observed variable *Y*. Let *R* = 1 if *Y* is observed and *R* = 0 if *Y* is missing. The MAR assumption states that, conditional on the observed data, missingness of *Y* does not depend on the unobserved data. This can be formulated as

Pr(R|Y,X)=Pr(R|X)

or equivalently as

Pr(Y|X,R=1)=Pr(Y|X,R=0).

#### Constructing the imputation model

The imputation model should include all of the variables in the analysis model(s) of interest as well as any variable that is a significant predictor of both the HIV test result and missingness of the HIV test result (12).

We constructed 4 imputation models of increasing complexity for the HIV test result variable under the MAR assumption. Model A was a logistic regression of HIV test results on age and region only. Model B included the variables in model A plus active pulmonary TB. Model C included the variables in model B plus current TB treatment, past TB treatment, household wealth index, educational attainment, marital status, diabetes status, smoking status, alcohol consumption, hunger in past 3 months, household crowding, circumcision status (males only), current cough, persistent cough for more than 2 weeks, current chest pain, current fever, current night sweats, current shortness of breath, and unintentional weight loss in past month. Model D included all of the variables in model C plus the auxiliary HIV self-report variable. Because the risk factors for a positive HIV test and for HIV test refusal varied by sex and also because our analysis models contained an interaction term for age by sex, we imputed missing HIV status values for men and women separately in all 4 models. We created *M* = 25 imputed data sets under each imputation model using the mice package in R (13). Our imputation procedure did not account for clustering by census enumeration area or household because this had little impact on inference in complete-case analyses (data not shown). We did not include any additional interaction terms in the imputation model.

### Multiple imputation under MNAR using delta adjustment

Multiple imputation with delta adjustment offers a transparent and flexible means by which to impute univariate data under general MNAR mechanisms, and thus to assess sensitivity to departures from MAR. Inspired by original proposals in Rubin (14), it has previously been used by van Buuren et al. (15) and implemented for a variety of variable types in the R package SensMice by Resseguier et al. (16). Further examples can be found in Carpenter and Kenward (17, 18).

After fitting an imputation model for the incomplete variable *Y* under MAR, implementation of the delta-adjustment procedure involves adding a fixed quantity δ to the linear predictor before imputing missing data using the updated model. As such, it is a simple type of pattern-mixture model. When *Y* is binary and the missing data are imputed using a logistic regression model, δ represents the difference in the log-odds of *Y* = 1 for individuals with missing *Y* values compared with individuals with observed *Y* values. A simple imputation model under MAR is

$$\mathrm{logit}\left\{\mathrm{Pr}\left[Y=1|X\right]\right\}=\Phi_{0}+\Phi_{X}^{\prime }X,$$

and a corresponding imputation model under MNAR is given by

$$\mathrm{logit}\{\mathrm{Pr}\left[Y=1\right|X,R]\}=\Phi_{0}+\Phi_{X}^{\prime }X+\delta \left(1-R\right),$$

where *R* = 1 if *Y* is observed and *R* = 0 if *Y* is missing. Varying δ across a range of values, ideally elicited from a subject-matter expert, produces an analysis of sensitivity to departures from MAR.

#### Extending the procedure

The delta-adjustment procedure can be refined to allow the degree of departure from MAR to vary among individuals with missing *Y* values according to their values on another fully observed variable *Z*. Examples can be found in Moreno-Betancur and Chavance (19) and Liublinska and Rubin (20). If *Z* is a 4-level categorical variable, we impute under the following model:

$$\begin{array}{l} \mathrm{logit}\{\mathrm{Pr}[Y=1|X,Z,R]\}\\ =\Phi_{0}+{\Phi \prime }_{X}X+\Phi_{Z}Z+\delta_{1}I_{\left\{z=1\right\}}(1-R)\\ +\delta_{2}I_{\left\{z=2\right\}}(1-R)+\delta_{3}I_{\left\{z=3\right\}}(1-R)+\delta_{4}I_{\left\{z=4\right\}}(1-R). \end{array}$$

#### Choice of adjustment values

Our final choice of adjustment values was informed by findings from a study that used data from 3 consecutive, annual rounds of HIV counseling and testing in the Karonga District of Malawi between 2007 and 2010 to investigate patterns in refusal of HIV testing over time (6). Given the result of their last HIV test, this study provided estimates of the proportion of individuals self-reporting as HIV-positive and HIV-negative as well as the proportion that accepted or refused HIV testing at the next testing round. We adjusted these figures to take account of differences in testing behavior between the populations in the Malawi and ZAMSTAR studies. We used these estimates in conjunction with expert opinion and the observed ZAMSTAR data to obtain an appropriate set of sensitivity parameter values. Further details of our approach are provided in Web Appendix 1 (available at http://aje.oxfordjournals.org/), including illustrative probability trees (Web Figures 1 and 2). Example R code for implementing the imputation procedure is provided in Web Appendix 2.

### Parametric causal mediation analysis

Analyses assessing sensitivity to departures from MAR can be difficult to perform when the primary analysis is of a complex form that requires multiple subcomponent models to be fitted to the data. Multiple imputation is particularly well-suited to such situations. Here we demonstrate how the delta-adjustment procedure can be used to assess the impact of departures from MAR on estimates arising from a parametric causal mediation analysis. This analysis investigated whether part of the observed relationship between educational attainment and active pulmonary TB can be explained via HIV status. While we use the term “effect” throughout, as with any observational study, we cannot rule out the possibility of uncontrolled confounding, issues surrounding the exposure definition, and model misspecification.

Valeri and VanderWeele (3) presented an integrated framework for parametric mediation analysis that is valid in the presence of exposure-mediator interaction and allows for the outcome and mediator variables to be any combination of binary, categorical, continuous, or count. This extended previous work that considered only a binary outcome and a continuous mediator (21). The approach involves fitting 2 parametric regression models to the data: a regression of the outcome on the exposure, mediator, and other confounders and a regression of the mediator on exposure and other confounders. The exposure variable can take 2 or more levels. In our example, TB status was the outcome, HIV test result was the mediator, educational attainment was the exposure, and we fitted 2 logistic regression models. Our confounder set for this analysis contained age, sex, region, and an age by sex interaction, resulting in 48 observed covariate patterns. Primary education was used as the reference category for the educational-attainment exposure variable.

The Valeri and VanderWeele approach (3) decomposes the total effect of setting the exposure to level *a* rather than to level $a^{⁎}$ as the product of a natural direct effect (NDE) and a natural indirect effect (NIE) on the odds-ratio scale. Such a decomposition is often not possible using the standard approach of Baron and Kenny (22). The causal effects are identified assuming that there is no unobserved confounding of any of the outcome-exposure, outcome-mediator, or mediator-exposure relationships and that no confounder of the outcome-mediator relationship is associated with the exposure. While the latter assumption may not be satisfied in our setting, this does not affect our ability to illustrate the delta-adjustment method. Although the NDE does not vary when there is no exposure-mediator interaction, in general the NDE, NIE, and total effect depend on the values of the confounding variables. Standard errors for these quantities can be obtained via bootstrapping or the multivariate delta method (3). Further details are provided in Web Appendix 3.

We implemented the parametric causal mediation analysis within the multiple imputation framework as follows: We first fitted the regression models for the outcome and the mediator in each imputed data set and then pooled the resulting imputation-specific coefficient estimates and their variance-covariance matrices using Rubin's rules. Finally, we calculated the causal-effect estimates and their standard errors.

## RESULTS

### Risk factors for HIV infection and HIV test refusal

Odds ratios for a number of potential risk factors for HIV infection and HIV test refusal, stratified by sex and adjusted for age and region, are presented in Tables 1 and 2, respectively. Self-reported HIV status was strongly related to both a positive HIV test and HIV test refusal in this sample, and its distribution varied considerably by sex, age, and region (Web Table 1).

**Table 1.** Odds Ratios for Refusal of Human Immunodeficiency Virus Testing Among Zambian Adults^a^ According to Individual and Household Characteristics, Zambia–South Africa TB and AIDS Reduction Study, 2010

Characteristic	Men (*n* = 11,484)					Women (*n* = 22,314)				
	No. Who Refused Test	No. at Risk	%	OR^b^	95% CI	No. Who Refused Test	No. at Risk	%	OR^b^	95% CI
Age, years										
18–24	1,362	4,294	31.7	1.00	Referent	2,250	7,804	28.8	1.00	Referent
25–29	532	1,651	32.2	1.05	0.93, 1.19	1,307	4,150	31.5	1.13	1.04, 1.23
30–34	448	1,355	33.1	1.06	0.93, 1.21	977	2,916	33.5	1.24	1.13, 1.36
35–39	373	1,053	35.4	1.18	1.02, 1.36	650	1,934	33.6	1.23	1.11, 1.37
40–49	467	1,291	36.2	1.19	1.04, 1.35	786	2,533	31.0	1.10	0.99, 1.21
>50	618	1,840	33.6	1.08	0.96, 1.21	935	2,977	31.4	1.09	1.00, 1.20
Region and TB risk										
Rural, low ARTI	1,223	3,564	34.3	1.00	Referent	2,138	6,232	34.3	1.00	Referent
Urban, low ARTI	1,053	2,616	40.3	1.29	1.17, 1.44	1,749	4,723	37.0	1.13	1.04, 1.22
Urban (not Lusaka), high ARTI	831	2,303	36.1	1.08	0.97, 1.21	1,408	4,262	33.0	0.95	0.87, 1.03
Lusaka, high ARTI	693	3,001	23.1	0.58	0.52, 0.65	1,610	7,097	22.7	0.56	0.52, 0.61
Self-reported HIV status										
HIV-negative	1,255	4,296	29.2	0.76	0.70, 0.83	3,249	11,650	27.9	0.80	0.75, 0.85
HIV-positive	235	562	41.8	1.25	1.05, 1.50	721	1,921	37.5	1.21	1.09, 1.35
Refused to disclose result	131	293	44.7	1.45	1.14, 1.84	325	718	45.3	1.58	1.35, 1.85
Never tested	2,179	6,333	34.4	1.00	Referent	2,610	8,025	32.5	1.00	Referent
Active pulmonary TB										
Yes	35	92	38.0	1.27	0.83, 1.95	28	99	28.3	0.90	0.58, 1.40
No	3,765	11,392	33.0	1.00	Referent	6,877	22,215	31.0	1.00	Referent
Educational attainment^c^										
None	85	276	30.8	1.14	0.87, 1.50	411	1,411	29.1	1.08	0.95, 1.23
Primary	727	2,589	28.1	1.00	Referent	2,258	8,264	27.3	1.00	Referent
Lower secondary	833	2,824	29.5	1.10	0.98, 1.24	1,802	5,871	30.7	1.18	1.10, 1.28
Upper secondary	1,544	4,366	35.4	1.45	1.29, 1.62	1,804	5,282	34.2	1.42	1.31, 1.53
College/university	611	1,429	42.8	1.83	1.59, 2.10	630	1,486	42.4	1.83	1.63, 2.05

Abbreviations: ARTI, annual risk of tuberculosis infection; CI, confidence interval; HIV, human immunodeficiency virus; OR, odds ratio; TB, tuberculosis.

^a^ Participants responded to a 2010 survey on the prevalence of TB and HIV and had an evaluable TB sputum sample.

^b^ Adjusted for age and region.

^c^ Educational attainment according to grade level was defined as follows: primary, less than grade 8; lower secondary, grade 8 or 9; and upper secondary, grade 10, 11, or 12.

**Table 2.** Odds Ratios for Having a Positive Human Immunodeficiency Virus Test Result Among Zambian Adults^a^ According to Individual and Household Characteristics, Zambia–South Africa TB and AIDS Reduction Study, 2010

Characteristic	Men (*n* = 7,684)					Women (*n* = 15,409)				
	No. With Positive Test	No. at Risk	%	OR^b^	95% CI	No. With Positive Test	No. at Risk	%	OR^b^	95% CI
Age, years										
18–24	77	2,932	2.6	1.00	Referent	611	5,554	11.0	1.00	Referent
25–29	143	1,119	12.8	5.46	4.09, 7.27	672	2,843	23.6	2.50	2.21, 2.82
30–34	195	907	21.5	10.23	7.76, 13.50	589	1,939	30.4	3.55	3.12, 4.04
35–39	192	680	28.2	15.03	11.33, 19.93	435	1,284	33.9	4.21	3.65, 4.87
40–49	230	824	27.9	14.77	11.23, 19.42	482	1,747	27.6	3.14	2.74, 3.59
>50	125	1,222	10.2	4.39	3.27, 5.88	229	2,042	11.2	1.06	0.90, 1.24
Region and TB risk										
Rural, low ARTI	236	2,341	10.1	1.00	Referent	588	4,094	14.4	1.00	Referent
Urban, low ARTI	261	1,563	16.7	1.96	1.61, 2.39	750	2,974	25.2	2.01	1.78, 2.28
Urban (not Lusaka), high ARTI	182	1,472	12.4	1.35	1.09, 1.67	603	2,854	21.1	1.61	1.42, 1.83
Lusaka, high ARTI	283	2,308	12.3	1.34	1.11, 1.63	1,077	5,487	19.6	1.47	1.32, 1.65
Self-reported HIV status										
HIV-negative	173	3,041	5.7	0.44	0.37, 0.54	831	8,401	9.9	0.47	0.43, 0.53
HIV-positive	317	327	96.9	181.84	95.2, 347.3	1,166	1,200	97.2	147.95	104.1, 210.3
Refused to disclose result	34	162	21.0	1.89	1.25, 2.88	166	393	42.2	3.10	2.48, 3.87
Never tested	438	4,154	10.5	1.00	Referent	855	5,415	15.8	1.00	Referent
Active pulmonary TB										
Yes	26	57	45.6	4.93	2.79, 8.73	35	71	49.3	3.74	2.29, 6.10
No	936	7,627	12.3	1.00	Referent	2,983	15,338	19.4	1.00	Referent
Educational attainment^c^										
None	31	191	16.2	1.06	0.69, 1.61	153	1,000	15.3	0.77	0.64, 0.94
Primary	301	1,862	16.2	1.00	Referent	1,333	6,006	22.2	1.00	Referent
Lower secondary	266	1,991	13.4	0.99	0.82, 1.20	897	4,069	22.0	1.03	0.93, 1.14
Upper secondary	262	2,822	9.3	0.79	0.66, 0.96	503	3,478	14.5	0.74	0.65, 0.83
College/university	102	818	12.5	0.71	0.55, 0.92	132	856	15.4	0.60	0.49, 0.73

Abbreviations: ARTI, annual risk of tuberculosis infection; CI, confidence interval; HIV, human immunodeficiency virus; OR, odds ratio; TB, tuberculosis.

^a^ Participants responded to a 2010 survey on the prevalence of TB and HIV, had an evaluable TB sputum sample, and agreed to be tested for HIV.

^b^ Adjusted for age and region.

^c^ Educational attainment according to grade level was defined as follows: primary, less than grade 8; lower secondary, grade 8 or 9; and upper secondary, grade 10, 11, or 12.

### Sensitivity analyses

We first assumed that the degree of departure from MAR was identical for all individuals with missing HIV serological values. In this case, δ represented the difference in the log-odds of a positive HIV test result for individuals with missing HIV test results compared with individuals with observed HIV test results. We considered a range of values from exp(δ) = 1.0 to exp(δ) = 5.0.

We then explored the impact of allowing the degree of departure from MAR to vary according to self-reported HIV status *Z*. We let δ_1_, δ_2_, δ_3_, and δ_4_ capture the degree of departure from MAR for individuals who self-reported as HIV-negative, who self-reported as HIV-positive, who refused to disclose their most recent test result, and who reported that they had never been tested for HIV, respectively. The values chosen for δ_1_, δ_2_, δ_3_, and δ_4_ (summarized in Table 3) captured our beliefs about the missing-data mechanism, assuming that no individual failed to report having had a prior HIV test. Missingness for individuals who self-reported as HIV-negative was believed to be MNAR (exp(δ_1_) > 1), because in addition to those who tested negative at their last test, this group includes individuals who know or suspect that they are HIV-positive but prefer to report as HIV-negative. Conversely, missingness for individuals who self-reported as HIV-positive was believed to be MAR (exp(δ_1_) = 1). Missingness for individuals who refused to disclose their status was believed to be strongly MNAR (exp(δ_3_) > 1), while missingness for individuals who reported that they had never previously been tested for HIV was believed to be MAR or weakly MNAR (exp(δ_4_) of close to 1).

**Table 3.** Summary of Analyses Assessing Sensitivity to Departures From the Missing-at-Random Assumption

Self-Reported HIV Status	Assumed Refusal Type	Sensitivity Parameter	Range of Values
HIV-negative	Strongly MNAR	δ_1_	ln(1.00,1.25,1.33,1.50,1.67,2.00,2.50,3.00, 4.00,5.00)
HIV-positive	MAR	δ_2_	ln(1.00)
Refused to disclose result	Strongly MNAR	δ_3_	ln(1.00,1.25,1.33,1.50,1.67,2.00,2.50,3.00, 4.00,5.00)
Never tested	Weakly MNAR	δ_4_	ln(0.75,0.80,1.00,1.25,1.33)

Abbreviations: HIV, human immunodeficiency virus; MAR, missing at random; MNAR, missing not at random.

### Estimation of HIV prevalence

Table 4 presents estimates of HIV prevalence from a complete-case analysis, best- and worst-case analyses (in which all missing HIV test values were imputed as 0 or 1, respectively), and the 4 alternative multiple-imputation analyses under MAR. Table 5 presents estimates of HIV prevalence from a selected subset of multiple-imputation analyses under MNAR based on imputation model D. The estimates from complete-case analysis were systematically lower than those produced by multiple imputation under MAR, while imputation models A, B, and C produced very similar estimates of the overall HIV prevalence. Including self-reported HIV status in the imputation model resulted in an increased estimate of the overall HIV prevalence.

**Table 4.** Estimates of the Prevalence of Human Immunodeficiency Virus Among Zambian Adults^a^, Zambia–South Africa TB and AIDS Reduction Study, 2010

Analysis Method	Overall (*n* = 33,798)		Men (*n* = 11,484)		Women (*n* = 22,314)		Reported HIV-Negative Result (*n* = 15,946)		Reported HIV-Positive Result (*n* = 2,483)		Refused to Disclose HIV Test Result (*n* = 1,011)		Never Tested (*n* = 14,358)	
	%	SE	%	SE	%	SE	%	SE	%	SE	%	SE	%	SE
Complete-case analysis^b^	17.1	0.2	12.5	0.4	19.4	0.3	8.8	0.3	97.0	0.4	36.0	2.0	13.3	0.3
Worst-case imputation^c^	43.5	0.3	41.5	0.5	44.5	0.3	34.6	0.4	98.2	0.3	65.1	1.5	42.4	0.4
Best-case imputation^d^	11.7	0.2	8.3	0.3	13.4	0.2	6.3	0.2	59.7	1.0	19.6	1.2	8.8	0.2
Multiple imputation under MAR														
Model A^e^	17.4	0.3	12.8	0.4	19.8	0.3	11.4	0.3	69.1	1.1	29.1	1.8	14.3	0.3
Model B^f^	17.4	0.3	12.8	0.4	19.8	0.3	11.4	0.3	69.3	1.1	29.1	1.7	14.3	0.4
Model C^g^	17.5	0.2	12.8	0.4	19.9	0.3	11.1	0.3	74.4	1.1	30.1	1.6	13.9	0.3
Model D^h^	18.1	0.2	13.4	0.4	20.6	0.3	8.8	0.3	96.9	0.4	36.0	2.0	13.6	0.3

Abbreviations: HIV, human immunodeficiency virus; MAR, missing at random; SE, standard error; TB, tuberculosis.

^a^ Participants responded to a 2010 survey on the prevalence of TB and HIV and had an evaluable TB sputum sample.

^b^*n* = 23,093.

^c^ All missing HIV test result values were imputed as positive.

^d^ All missing HIV test result values were imputed as negative.

^e^ Imputation model included age and region only.

^f^ Imputation model included age, region, and active pulmonary TB only.

^g^ Imputation model included age, region, active pulmonary TB, household wealth index, educational attainment, current TB treatment, past TB treatment, marital status, diabetes status, smoking status, alcohol consumption, hunger in past 3 months, household crowding, circumcision status (males only), current cough, persistent cough for more than 2 weeks, current chest pain, current fever, current night sweats, current shortness of breath, and unintentional weight loss in past month.

^h^ Imputation model included all variables in model C with the addition of self-reported HIV status.

**Table 5.** Estimates of the Prevalence of Human Immunodeficiency Virus Among Zambian Adults^a^ Arising From an Analysis of Sensitivity to Departures From the Missing-at-Random Assumption, Zambia–South Africa TB and AIDS Reduction Study, 2010

Multiple Imputation Under MNAR^b^				Overall (*n* = 33,798)		Men (*n* = 11,484)		Women (*n* = 22,314)		Reported HIV-Negative Result (*n* = 15,946)		Reported HIV-Positive Result (*n* = 2,483)		Refused to Disclose HIV Test Result (*n* = 1,011)		Never Tested (*n* = 14,358)	
exp(δ_1_)^c^	exp(δ_2_)^d^	exp(δ_3_)^e^	exp(δ_4_)^f^	%	SE	%	SE	%	SE	%	SE	%	SE	%	SE	%	SE
1.0	1.0	1.0	1.0	18.1	0.2	13.4	0.4	20.6	0.3	8.8	0.3	96.9	0.4	36.0	2.0	13.6	0.3
2.5	1.0	2.5	1.0	19.7	0.3	14.3	0.4	22.4	0.3	11.5	0.3	96.9	0.4	44.1	2.1	13.6	0.3
2.5	2.5	2.5	2.5	21.4	0.3	16.3	0.4	24.1	0.4	11.5	0.3	97.6	0.3	44.1	2.1	17.7	0.4
5.0	1.0	2.5	1.0	21.1	0.3	15.2	0.4	24.2	0.4	14.6	0.4	96.9	0.4	44.1	2.1	13.6	0.3
5.0	1.0	5.0	1.0	21.3	0.3	15.3	0.4	24.4	0.4	14.6	0.4	96.9	0.4	50.1	2.0	13.6	0.3
5.0	5.0	5.0	5.0	24.9	0.3	19.4	0.5	27.7	0.4	14.6	0.4	97.9	0.3	50.1	2.0	21.8	0.5

Abbreviations: HIV, human immunodeficiency virus; MAR, missing at random; MNAR, missing not at random; SE, standard error; TB, tuberculosis.

^a^ Participants responded to a 2010 survey on the prevalence of TB and HIV and had an evaluable TB sputum sample.

^b^ Imputation model included age, region, active pulmonary TB, household wealth index, educational attainment, current TB treatment, past TB treatment, marital status, diabetes status, smoking status, alcohol consumption, hunger in past 3 months, household crowding, circumcision status (males only), current cough, persistent cough for more than 2 weeks, current chest pain, current fever, current night sweats, current shortness of breath, unintentional weight loss in past month, and self-reported HIV status.

^c^ δ_1_ is the degree of departure from MAR for individuals who self-reported as HIV-negative.

^d^ δ_2_ is the degree of departure from MAR for individuals who self-reported as HIV-positive.

^e^ δ_3_ is the degree of departure from MAR for individuals who refused to disclose the result of their most recent HIV test.

^f^ δ_4_ is the degree of departure from MAR for individuals who reported having no prior HIV tests.

In MNAR analyses, estimates of the overall HIV prevalence varied from 18.1% under MAR (exp(δ) = l.0) to 24.9% when exp(δ) = 5.0. Allowing the degree of departure from MAR to vary according to self-reported HIV status, as captured by the group-specific δ_*j*_ values, was associated with more subtle differences in the estimates of HIV prevalence than applying a common δ value to all participants with missing HIV test result data (Table 5). This is further illustrated by the filled contour plot in Web Figure 3, which presents overall and sex-stratified estimates of HIV prevalence by group-specific δ_*j*_ values.

### Parametric causal mediation analysis

#### Complete-case and MAR analyses

We first fitted the 2 subcomponent logistic regression models to the data. Because there was no evidence of exposure-mediator interaction, we refitted the outcome regression model, omitting this term. Estimated odds ratios from each subcomponent model arising from complete-case, best-case, worst-case, and multiple-imputation-under-MAR analyses are reported in Web Tables 2 and 3. While HIV status partly mediated the effects of having upper-secondary or college/university education (compared with primary education) on active pulmonary TB across a majority of covariate patterns, there was no evidence that HIV status mediated the effects of having lower-secondary education. The NIE on active pulmonary TB of having no education compared with having primary education was in the opposite direction of the NDE for all covariate patterns, indicating a lack of mediation. Accounting for missing data via multiple imputation under MAR did not produce a qualitative change in inference regarding mediation. A representative set of causal-effect estimates and 95% confidence intervals for one covariate pattern is presented in Table 6.

**Table 6.** Estimates of the Natural Direct Effect, Natural Indirect Effect, and Total Effect of Educational Attainment on the Odds of Active Pulmonary Tuberculosis as Mediated by Human Immunodeficiency Virus Status Among Selected Zambian Women^a^, Zambia–South Africa TB and AIDS Reduction Study, 2010

Education and Type of Effect	Complete-Case Analysis^b^		Best-Case Analysis^c^		Worst-Case Analysis^d^		Multiple Imputation Under MAR^e^	
	OR	95% CI	OR	95% CI	OR	95% CI	OR	95% CI
None vs. primary education								
Natural direct effect	1.88	0.97, 3.64	1.43	0.77, 2.66	1.40	0.75, 2.59	1.45	0.78, 2.70
Natural indirect effect	0.94	0.86, 1.03	0.94	0.88, 1.01	1.00	0.94, 1.05	0.95	0.92, 0.99
Total effect	1.76	0.90, 3.44	1.35	0.72, 2.51	1.39	0.75, 2.59	1.38	0.74, 2.58
Lower-secondary vs. primary education								
Natural direct effect	0.71	0.45, 1.11	0.78	0.54, 1.12	0.76	0.52, 1.09	0.77	0.54, 1.12
Natural indirect effect	1.00	0.96, 1.05	0.99	0.95, 1.02	1.02	0.99, 1.05	1.01	0.99, 1.03
Total effect	0.71	0.45, 1.12	0.77	0.53, 1.11	0.77	0.53, 1.11	0.78	0.54, 1.12
Upper-secondary vs. primary education								
Natural direct effect	0.67	0.41, 1.08	0.71	0.48, 1.04	0.65	0.44, 0.95	0.73	0.50, 1.07
Natural indirect effect	0.92	0.87, 0.97	0.90	0.86, 0.94	1.03	1.00, 1.07	0.95	0.92, 0.97
Total effect	0.61	0.38, 1.00	0.64	0.43, 0.94	0.67	0.45, 0.98	0.69	0.47, 1.01
College/university vs. primary education								
Natural direct effect	0.33	0.12, 0.93	0.29	0.13, 0.64	0.24	0.11, 0.53	0.30	0.14, 0.66
Natural indirect effect	0.87	0.80, 0.95	0.84	0.77, 0.90	1.06	1.01, 1.11	0.92	0.88, 0.95
Total effect	0.29	0.10, 0.81	0.24	0.11, 0.53	0.26	0.12, 0.57	0.27	0.12, 0.60

Abbreviations: CI, confidence interval; HIV, human immunodeficiency virus; OR, odds ratio; TB, tuberculosis.

^a^ Participants responded to a 2010 survey on the prevalence on TB and HIV and had an evaluable TB sputum sample. This table shows results for female participants in Zambia, aged 25–29 years, living in urban communities with low annual risk of tuberculosis infection.

^b^*n* = 23,093.

^c^ All missing HIV test result values were imputed as positive.

^d^ All missing HIV test result values were imputed as negative.

^e^ Imputation model included age, region, active pulmonary TB, household wealth index, educational attainment, current TB treatment, past TB treatment, marital status, diabetes status, smoking status, alcohol consumption, hunger in past 3 months, household crowding, circumcision status (males only), current cough, persistent cough for more than 2 weeks, current chest pain, current fever, current night sweats, current shortness of breath, unintentional weight loss in past month, and self-reported HIV status.

#### Analyses assessing sensitivity to departure from the MAR assumption

**Figure 1.**
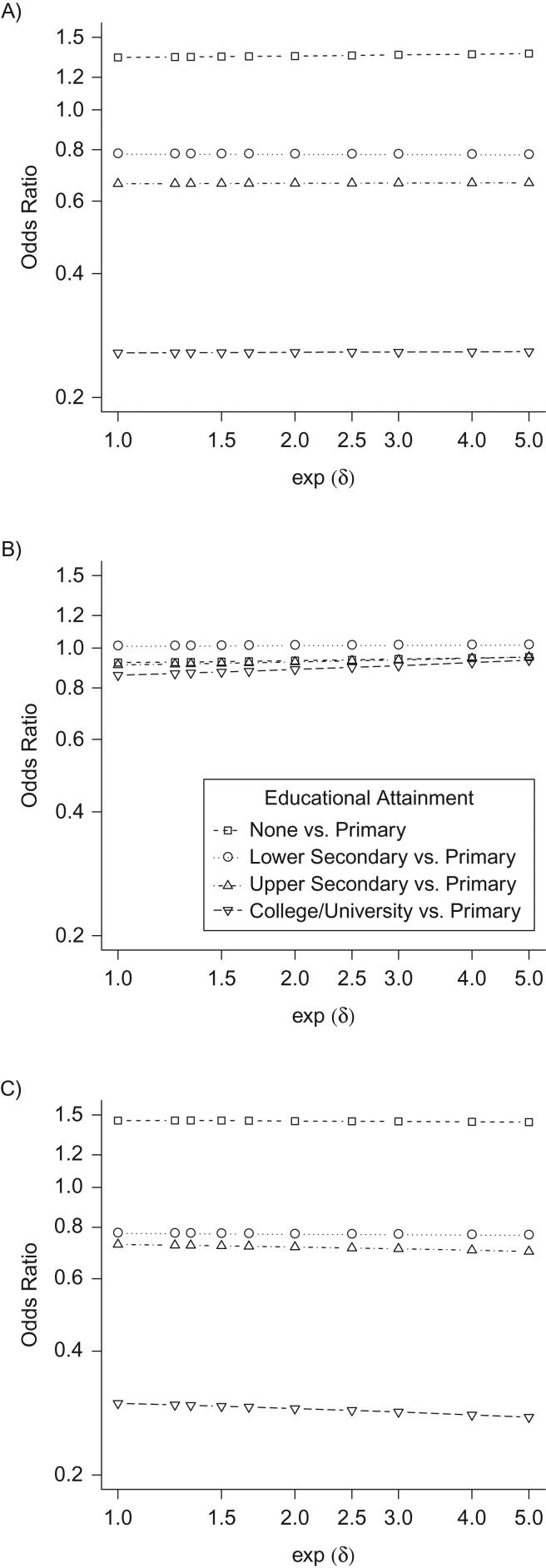
Estimated causal effects of educational attainment on active pulmonary tuberculosis (TB) as mediated by human immunodeficiency virus status, according to degree of departure (δ = δ_1_ = δ_2_ = δ_3_ = δ_4_) from the missing-at-random assumption in the HIV test result variable, Zambia–South Africa TB and AIDS Reduction Study, 2006–2010. A) Natural direct effect; B) natural indirect effect; C) total effect. These analyses are of data from female Zambian adults aged 25–29 years, living in urban communities with a low annual risk of TB infection.

Estimates of the average NDE for each level of the educational-attainment exposure variable were insensitive to departures from the MAR assumption across a majority of covariate patterns. While estimates of the NIE of having a college/university education exhibited moderate sensitivity to departures from MAR across all covariate patterns, estimates of the NIE for the remaining exposure levels exhibited little sensitivity. Sensitivity of the average NIE and total effect to departures from MAR was primarily attributable to sensitivity of the coefficient estimate for the educational-attainment exposure in the model for the mediator (Web Figure 4). In general, accounting for possible violation of the MAR assumption was not associated with a qualitative change in inference regarding mediation. A sensitivity analysis for the covariate pattern shown in Table 6 is presented in Figure 1.

## DISCUSSION

In this study, we reviewed multiple imputation with the delta-adjustment procedure and demonstrated how it can be used to impute data under general MNAR mechanisms, thus facilitating analysis of sensitivity to departures from the MAR assumption. We applied the approach to data from a survey on TB/HIV prevalence, conducted as part of the ZAMSTAR Study, assessing the impact of departures from MAR on HIV prevalence and causal-effect estimates in 2 types of sensitivity analysis. The first sensitivity analysis assumed that the degree of departure from MAR was the same for all individuals with missing HIV serological values, while the second assumed that the degree of departure from MAR varied according to an individual's self-reported HIV status. Although we assumed that the degree of departure from MAR for individuals with missing HIV test result values did not vary according to TB status or educational attainment, sensitivity analyses exploring the impact of such dependencies could be performed in an identical fashion.

Our approach to sensitivity analysis produces a range of inferences by varying the sensitivity parameters across a range of plausible values. This allows the investigator to explore how the inference changes according to the assumption placed on the missing-data mechanism. A possible alternative, attractive to policy-makers, provides a single inference by placing informative prior distributions on the sensitivity parameters in a fully Bayesian analysis (23). Recently developed multiple-model multiple-imputation approaches (24, 25) can be used to approximate such analyses within the multiple-imputation framework.

We acknowledge that elicitation of the sensitivity parameter values can represent a significant challenge in many applied research settings. In situations where there is a clear hypothesis to be tested—for example, determining whether HIV prevalence has fallen below a specified value—it can be easier to conduct a tipping-point analysis (20, 26). In this approach, the investigator varies the sensitivity parameters across a large range of values in order to determine a set of values for which there is a qualitative change in inference. The investigator must then evaluate whether this set of values is plausible for the data at hand and thus whether the results of their analyses are sensitive to departures from MAR. Improved tools for the elicitation of sensitivity parameters are needed if MNAR methods are to enjoy routine use among applied researchers.

Multiple imputation offers a rigorous approach by which to incorporate auxiliary information on self-reported HIV status into analyses based on partially observed HIV serological analysis. Exploiting auxiliary information on self-reported HIV status produced estimates of overall and subgroup-specific HIV prevalence with greater face validity when it was included as a variable in the imputation model and also allowed us to perform more sophisticated analyses of sensitivity to departures from the MAR assumption. Future population-based studies should continue to collect information on self-reported HIV status in addition to testing for HIV, especially in settings with high rates of prior testing. Seeking more information on past HIV-testing behavior (for example, the date of the most recent HIV test) or beliefs about status if never tested would also be valuable. For example, we encountered some difficulty in selecting an appropriate range of delta values for the never-tested subgroup. This group is likely to contain a mixture of individuals at quite different levels of risk of HIV infection. Some individuals might not have access to testing, some might refuse testing because they believe themselves to be at very low risk, and others might refuse testing because they believe themselves to be at high risk and fear disclosure. In the absence of further information about the composition of this subgroup, it may be reasonable to consider a larger range of values for the degree of departure from MAR than was presented here—for example, from exp(δ_4_) = 0.5 to exp(δ_4_) = 2.0.

Our causal-effect estimates exhibited marked insensitivity to departures from MAR. Nevertheless, the validity of these estimates depends critically on the set of identifying restrictions detailed earlier and on the assumption that the 2 component parametric models are correctly specified. While we are confident that we have captured the most important confounders of the outcome-mediator, outcome-exposure, and mediator-exposure relationships—and that the confounders of the outcome-mediator relationship for which we adjusted are not associated with the exposure—the impact of violations of these assumptions could be explored in further sensitivity analyses. For example, Tchetgen Tchetgen and Phiri (27) and Naimi (28) have derived bounds for natural effects when the exposure is associated with one or more confounders of the outcome-mediator relationship. Furthermore, some readers may not agree that educational attainment constitutes a well-defined counterfactual cause (29); further discussion of this perspective is provided in Web Appendix 3.

While we have focused on an example with a single incomplete variable, we note that delta-adjustment procedures can also be used to adjust for missing data in longitudinal clinical trials subject to dropout (19, 30). Furthermore, while some authors (15, 16) have attempted to perform delta adjustment in conjunction with the chained-equations algorithm, at present this approach lacks a strong theoretical foundation and thus should be used with caution.

In conclusion, multiple imputation with delta adjustment offers a transparent and flexible means to perform analyses of sensitivity to departures from the MAR assumption in the presence of a single incomplete variable. While appropriate for use in conjunction with all types of univariable and multivariable analysis, this method may represent a particularly important tool for sensitivity analysis in contexts such as mediation analysis where multiple subcomponent models must be fitted to the data.

## Supplementary Material

Web Material

## Abbreviations

AIDS: acquired immune deficiency syndrome
HIV: human immunodeficiency virus
MAR: missing at random
MNAR: missing not at random
NDE: natural direct effect
NIE: natural indirect effect
TB: tuberculosis
ZAMSTAR: Zambia–South Africa TB and AIDS Reduction
