# Supplementary material for: Analyses of Sensitivity to the Missing-at-Random Assumption Using Multiple Imputation With Delta Adjustment: Application to a Tuberculosis/HIV Prevalence Survey With Incomplete HIV-Status Data
Source: Am J Epidemiol. 2017 Jan 10;185(4):304–15. doi: 10.1093/aje/kww107 (PMC5860630; doi:10.1093/aje/kww107)
Supplement: Web Material [file kww107leacywebmaterialfinalrevised.pdf]

# Web Material for

# "Sensitivity Analyses to the Missing at Random Assumption Using Multiple Imputation with delta-adjustment: Application to a Tuberculosis/HIV Prevalence Survey with Incomplete HIV-Status Data"

Finbarr P. Leacy*

Sian Floyd

Tom A. Yates

Ian R. White

*Email: finbarrleacy@rcsi.ie

## WEB APPENDIX 1: SELECTING SENSITIVITY PARAMETER VALUES

Plausible values for the probabilities in web figure 1 can be informed by the observed data, the published literature, pilot studies and expert opinion.

- Suppose the total population is of size **N**.
- Let π denote the proportion of the population with a previous HIV test.
- Suppose that no individual fails to report a prior HIV test.
- Suppose that individuals with a prior HIV test are asked to disclose the result of their most recent test.
- If we assume that prior testing is independent of true HIV status, then **p=q**.
- If we assume that no individual who tested negative at their most recent test will self-report as HIV positive, then $\mathbf{s_1=0}$ and consequently **K=II=JJ=0**.

A range of plausible delta values can be derived by varying the values of these probabilities and/or relaxing one or more of these assumptions.

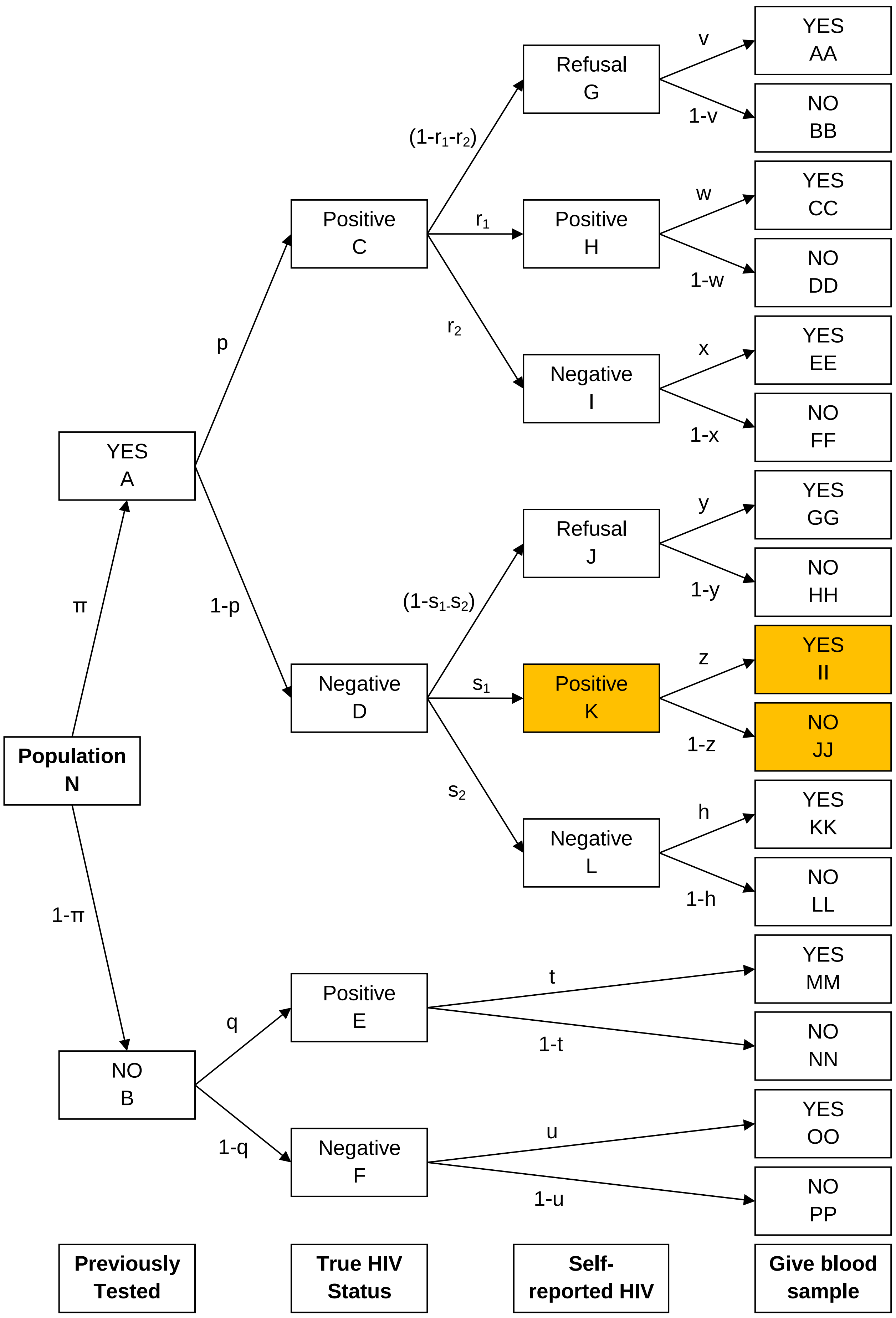


**Web Figure 1.** Probability tree used to derive group-specific sensitivity parameter values.

**Overall:**

HIV prevalence among those who give a blood sample = $\frac{(AA + CC + EE + MM)}{(AA + CC + EE + GG + II + KK + MM + OO)}$

HIV prevalence among those who do not give a blood sample = $\frac{(BB + DD + FF + NN)}{(BB + DD + FF + HH + JJ + LL + NN + PP)}$

$$OR = \frac{(AA + CC + EE + MM)(HH + JJ + LL + PP)}{(GG + II + KK + OO)(BB + DD + FF + NN)} = \exp(\delta)$$

**Among those who self-report as HIV-negative:**

HIV prevalence among those who give a blood sample = $\frac{EE}{(EE + KK)}$

HIV prevalence among those who do not give a blood sample = $\frac{FF}{(FF + LL)}$

$$OR = \frac{(EE)(LL)}{(FF)(KK)} = \exp(\delta_1)$$

**Among those who self-report as HIV-positive:**

HIV prevalence among those who give a blood sample = $\frac{CC}{(CC + II)}$

HIV prevalence among those who do not give a blood sample = $\frac{DD}{(DD + JJ)}$

$$OR = \frac{(CC)(JJ)}{(II)(DD)} = \exp(\delta_2)$$

If we further assume that $s_1=0$, then $II=JJ=0$ and OR = $\exp(\delta_2)$ = 1.0.

**Among those who refuse to disclose their status:**

HIV prevalence among those who give a blood sample = $\frac{AA}{(AA + GG)}$

HIV prevalence among those who do not give a blood sample = $\frac{BB}{(BB + HH)}$

$$OR = \frac{(AA)(HH)}{(GG)(BB)} = \exp(\delta_3)$$

**Among those with no prior test:**

HIV prevalence among those who give a blood sample = $\frac{MM}{(MM + OO)}$

HIV prevalence among who do not give a blood sample = $\frac{NN}{(NN + PP)}$

$$OR = \frac{(MM)(PP)}{(OO)(NN)} = \exp(\delta_4)$$

**Among those with a previous test:**

HIV prevalence among those who give a blood sample = $\frac{(AA + CC + EE)}{(AA + CC + EE + GG + II + KK)}$

HIV prevalence among those who do not give a blood sample = $\frac{(BB + DD + FF)}{(BB + DD + FF + HH + JJ + LL)}$

$$OR = \frac{(AA + CC + EE)(HH + JJ + LL)}{(GG + II + KK)(BB + DD + FF)}$$

**Worked Example**

Individuals participating in the ZAMSTAR TB/HIV prevalence survey were asked the following series of questions:

1. Do you know your status?

   IF YES:

2. Are you willing to disclose your status?

   IF YES:

4. Are you HIV-positive or HIV-negative?

Plausible values for the probabilities in the probability tree shown in web figure 2 were informed by data collected in the ZAMSTAR trial itself (1), assumptions detailed in (2), and findings from a study investigating patterns in HIV refusal over time in the Karonga District of Malawi (3). We derive delta values for the entire adult population, but delta values could also be derived for males and females separately.

- Suppose we have a population of size **N=1000**.
- Suppose that the proportion of the population with a prior HIV test is $\boldsymbol{\pi = 0.575}$ (ZAMSTAR trial data)
- Assume that the HIV prevalence among individuals without a prior HIV test is $\mathbf{q = 0.15}$ (ZAMSTAR trial data)
- Assume that no individual fails to report a past HIV test.
- Assume that prior testing is independent of true HIV status (2), $\mathbf{p = q = 0.15}$.
- Among individuals without a prior test, assume that test acceptance is independent of true HIV status (2), $\mathbf{t = u = 0.67}$ (ZAMSTAR trial data).
- Among those who are truly HIV-positive and have previously been tested, $\mathbf{r_1 = 0.7}$ self-report as HIV-positive (ZAMSTAR trial data), $\mathbf{r_2 = 0.2}$ report as HIV-negative, and $\mathbf{1 - r_1 – r_2 = 0.1}$ refuse to disclose their status.
- Among those who are truly HIV-negative and have previously been tested, $\mathbf{s_1 = 0}$ self-report as HIV-positive, $\mathbf{s_2 = 0.95}$ report as HIV-negative (3), and $\mathbf{1 - s_1 – s_2 = 0.05}$ refuse to disclose their status.
- Among those who are truly HIV-positive, have previously been tested and self-report as HIV-positive, $\mathbf{w = 0.6}$ provide a blood sample for HIV testing (ZAMSTAR trial data).
- Among those who are truly HIV-positive, have previously been tested and self-report as HIV-negative, $\mathbf{x = 0.45}$ provide a blood sample for HIV testing (3).
- Among those who are truly HIV-positive, have previously been tested but refuse to disclose their status, $\mathbf{v = 0.45}$ provide a blood sample for HIV testing (expert opinion based on ZAMSTAR trial data).

- Among those who are truly HIV-negative, have previously been tested and self-report as HIV-negative, **h = 0.8** provide a blood sample for HIV testing (3).
- Among those who are truly HIV-negative, have previously been tested but refuse to disclose their status, **y = 0.6** provide a blood sample for HIV testing (expert opinion based on ZAMSTAR trial data).

As mentioned previously, we can derive a range of plausible delta values by varying the values of these probabilities and/or relaxing one or more of the assumptions made.

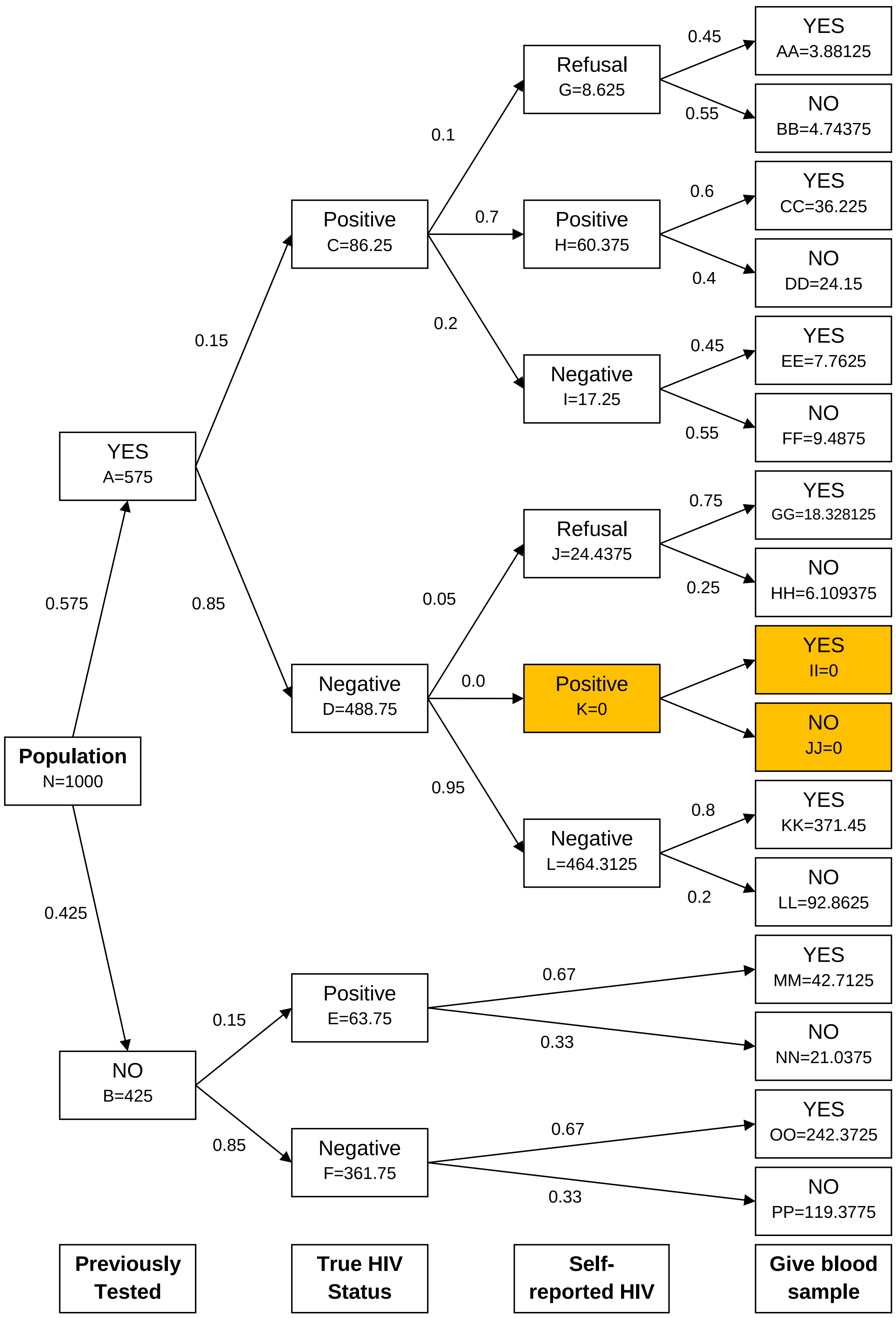


**Web Figure 2.** Probability tree used to derive group-specific sensitivity parameter values in the worked example.

**Overall:**

HIV prevalence among those who give a blood sample = 90.58125/722.731875 = 0.125

HIV prevalence among those who do not give a blood sample = 59.41875/277.768125 = 0.214

OR = exp(δ) = 1.90

**Among those who self-report as HIV-negative:**

HIV prevalence among those who give a blood sample = 7.7625/379.2125 = 0.020

HIV prevalence among those who do not give a blood sample = 9.4875/102.350 = 0.092

OR = exp($\delta_1$) = 5.06

**Among those who self-report as HIV-positive:**

HIV prevalence among those who give a blood sample = 1.0

HIV prevalence among those who do not give a blood sample = 1.0

OR = exp($\delta_2$) = 1.0

**Among those who refuse to disclose their status:**

HIV prevalence among those who give a blood sample = 3.88125/22.209375 = 0.175

HIV prevalence among those who do not give a blood sample = 4.74375/10.853125 = 0.439

OR = exp($\delta_3$) = 3.13

**Among those with no prior test:**

HIV prevalence among those who give a blood sample = 42.7125/285.085 = 0.150

HIV prevalence among who do not give a blood sample = 21.0375/140.415 = 0.150

OR = exp($\delta_4$) = 1.00

**Among those with a previous test:**

HIV prevalence among those who give a blood sample = 47.86875/437.646875 = 0.109

HIV prevalence among those who do not give a blood sample = 38.38125/137.353125 = 0.279

OR = 3.16

## WEB APPENDIX 2: EXAMPLE R CODE FOR IMPLEMENTING THE IMPUTATION PROCEDURE

```
# Load the mice package(4)
library(mice)

# Declare a new imputation function, mice.impute.logreg.sens
# This is a minor modification of the standard mice.impute.logreg
function contained in (4)
# The argument delta must be specified by the user
# delta is the difference in the log-odds of Y=1 for those with missing
Y values compared to those with observed Y values

mice.impute.logreg.sens <- function(y, ry, x, delta,...) {

     # The method consists of the following steps:
     # 1. Fit a logistic regression model, and find (bhat, V(bhat))
     # 2. Add delta to the linear predictor values
     # 3. Draw beta from N(bhat, V(bhat))
     # 4. Compute predicted scores for units with missing data,
     # logit^{-1}(X beta)
     # 5. Compare the score to a random (0,1) deviate, and impute.

     # 1. Fit a logistic regression model, and find (bhat, V(bhat))
     x <- cbind(1, as.matrix(x))
     expr <- expression(glm.fit(x[ry, ], y[ry],
                    family = binomial(link = logit))
     fit <- suppressWarnings(eval(expr))
     fit.sum <- summary.glm(fit)
     # Fitted coefficient values
     beta <- coef(fit)

     # 2. Add delta to the linear predictor values
     beta[1] <- beta[1] + delta

     # 3. Draw beta from N(bhat, V(bhat))
     rv <- t(chol(fit.sum$cov.unscaled))
     beta.star <- beta + rv %*% rnorm(ncol(rv))

     # 4. Compute predicted scores for units with missing data,
     # logit^{-1}(X beta)
     p <- 1/(1 + exp(-(x[!ry, ] %*% beta.star)))

     # 5. Compare the score to a random (0,1) deviate, and impute.
     vec <- (runif(nrow(p)) <= p)
     vec[vec] <- 1
     if (is.factor(y)) {
           vec <- factor(vec, c(0, 1), levels(y))
     }
  return(vec)
}
```

```
# Suppose we have a dataset with incomplete HIV status and fully
observed data on age, gender, region, TB status and self-reported HIV
status.
dta <- c("HIV", "age", "gender", "region", "TB", "selfrepHIV")

# Initialise arguments of the mice function
ini <- mice(data=dta, maxit=0)
# Default predictor matrix
pred <- ini$pred
# Default imputation function specification
meth <- ini$meth

# By default, all variables will be included in the imputation model for
HIV status
# We can remove variables from the imputation model for HIV as follows:
pred["HIV", c("selfrepHIV")] <- 0
# Specify the imputation function for HIV
meth["HIV"] <- "logreg.sens"

# Create imputed datasets
# data - incomplete dataset with missing values recorded as NA
# pred - predictor matrix
# meth - imputation function to be used
# m - Number of imputed datasets to be created
# maxit - Number of sampler iterations

imp <- mice(data=dta, pred=pred, meth=meth,
               m=25, maxit=1, delta=log(2.0))

# As per the mice package documentation, we can obtain an estimate of
the HIV prevalence as follows:

m <- imp$m
Q <- rep(NA, m)
U <- rep(NA, m)

# Estimate the HIV prevalence in each imputed dataset
for (i in 1:m){
     Q[i] <- mean(complete(imp, i)$HIV)
     U[i] <- var(complete(imp, i)$HIV) / nrow(dta) # (standard error)^2
}

# Combine the imputation-specific estimates using Rubin's Rules
pool.scalar(Q, U, n=nrow(dta), method = "rubin")
```

## WEB APPENDIX 3: PARAMETRIC CAUSAL MEDIATION ANALYSIS

Parametric mediation analysis involves fitting two parametric regression models to the data: a regression of the outcome on the exposure, mediator and other confounders and a regression of the mediator on exposure and other confounders. Using slightly different notation to Valeri and VanderWeele (5), suppose we have a binary outcome $Y$, a categorical exposure $A$ with two or more levels, a binary mediator $M$ and vector of confounders $\boldsymbol{C}$. The model for the outcome is given by

$$logit\{P[Y = 1|A = a, M = m, \boldsymbol{C} = \boldsymbol{c}]\} = \theta_0 + \theta_A a + \theta_M m + \theta_{AM} am + \boldsymbol{\theta}'_{\boldsymbol{C}}\boldsymbol{c}$$

and the model for the mediator is given by

$$logit\{P[M = 1|A = a, \boldsymbol{C} = \boldsymbol{c}]\} = \beta_0 + \beta_A a + \boldsymbol{\beta}_{\boldsymbol{C}'}\boldsymbol{c}$$

The natural direct effect (NDE), natural indirect effect (NIE) and the total effect (TE) are identified assuming that there is no unobserved confounding of the outcome-exposure, outcome-mediator or mediator-exposure relationships and that every confounder of the outcome-mediator relationship is unaffected by the exposure (5).

If these assumptions are satisfied, the average natural direct effect (NDE) of setting the exposure to level $a$ compared to level $a^*$, conditional on $\boldsymbol{C} = \boldsymbol{c}$, is given by

$$ORNDE \cong \frac{exp(\theta_A a)\{1 + exp(\theta_M m + \theta_{AM} a + \beta_0 + \beta_A a^* + \boldsymbol{\beta}'_{\boldsymbol{C}}\boldsymbol{c})\}}{exp(\theta_A a^*)\{1 + exp(\theta_M m + \theta_{AM} a^* + \beta_0 + \beta_A a^* + \boldsymbol{\beta}'_{\boldsymbol{C}}\boldsymbol{c})\}}$$

In the absence of exposure-mediator interaction, $\theta_{AM} = 0$, this simplifies to

$$ORNDE \cong exp(\theta_A(a - a^*))$$

and does not depend on the values of the confounding variables.

The average natural indirect effect (NIE) of setting the exposure to level $a$ compared to level $a^*$, conditional on $\boldsymbol{C} = \boldsymbol{c}$, is given by

$$OR^{NIE} \cong \frac{\{1 + \exp(\beta_0 + \beta_A a^* + \boldsymbol{\beta}'_{\boldsymbol{C}}\boldsymbol{c})\}\{1 + \exp(\theta_M m + \theta_{AM} a + \beta_0 + \beta_A a + \boldsymbol{\beta}'_{\boldsymbol{C}}\boldsymbol{c})\}}{\{1 + \exp(\beta_0 + \beta_A a + \boldsymbol{\beta}'_{\boldsymbol{C}}\boldsymbol{c})\}\{1 + \exp(\theta_M m + \theta_{AM} a + \beta_0 + \beta_A a^* + \boldsymbol{\beta}'_{\boldsymbol{C}}\boldsymbol{c})\}}$$

Note that in this expression all four components of the numerator and denominator include terms for the confounding variables: thus estimates of the average natural indirect effect are conditional on the values taken by the confounding variables. Note also that sensitivity of this quantity to departures from MAR is primarily attributable to sensitivity of $\beta_A$.

The average natural total effect (TE) of setting the exposure to level $a$ compared to level $a^*$, conditional on $\boldsymbol{C} = \boldsymbol{c}$, is then given by

$$ORTE = OR^{NDE} OR^{NIE}$$

Standard errors for these three quantities can be obtained by bootstrapping or through application of the multivariate delta method (5).

### Interpretation of the natural direct, natural indirect and total effect odds ratios

Suppose that we wish to estimate the causal effects, as mediated by HIV status, of setting educational attainment to College/university rather than to Primary.

The average natural direct effect is the odds ratio of active pulmonary TB, conditional on $\boldsymbol{C} = \boldsymbol{c}$, for individuals with College/university educational attainment and HIV status set to what it would have been had their educational attainment been set to Primary compared to individuals with Primary educational attainment and their natural HIV status at this exposure level.

The average natural indirect effect is the odds ratio of active pulmonary TB, conditional on $\boldsymbol{C} = \boldsymbol{c}$, for individuals with College/university educational attainment and their natural HIV status at this exposure level compared to individuals with College/university educational attainment and HIV status set to what it would have been had their educational attainment been set to Primary.

The total effect is the odds ratio of active pulmonary TB, conditional on $\boldsymbol{C} = \boldsymbol{c}$, for individuals with College/university educational attainment compared to individuals with Primary educational attainment. It is the product of the average natural direct effect and the average natural indirect effect on the odds ratio scale.

### Identification and Interpretation of Controlled Direct Effects

The Valeri and VanderWeele framework can also be used to estimate controlled direct effects (CDE). Assuming that there is no unobserved confounding of the outcome-exposure or outcome-mediator relationships, the controlled direct effect of setting the exposure to level $a$ rather than level $a^*$ with the mediator held fixed at level $m$ is given by

$$ORCDE = \exp\{(\theta_A + \theta_{AM} m)(a - a^*)\}.$$

While the CDE is equal to the NDE in the absence of exposure-mediator interaction, it is important to recognise that this quantity is identified even when the exposure affects one or more confounders of the outcome-mediator relationship and carries a different causal interpretation. In our setting, the controlled direct effect is the odds ratio of active pulmonary TB for individuals with College/University educational attainment compared to individuals with Primary educational attainment where the HIV status of all individuals is set to level $m$.

## Educational Attainment as a Counterfactual Cause

There is some disagreement in the literature as to whether it is appropriate to consider educational attainment and other social determinants of health such as race and gender as well-defined causes under the potential outcomes framework (6, 7). This is because interventions based on such exposures could plausibly violate key assumptions of counterfactual consistency and no interference in some settings (6). Naimi et al. (7) have argued that the controlled direct effect (CDE) can represent a suitable target of inference in the presence of a modifiable mediator of the outcome-exposure relationship even when the exposure is not well-defined. For example, suppose we are willing to accept HIV status but not educational attainment as a counterfactual cause. These authors would interpret estimates of the CDE of educational attainment on active pulmonary TB obtained in the current study as the magnitude of educational disparity in the prevalence of active pulmonary TB that would remain were the investigator to intervene to equalise the distribution of HIV status across all categories of educational attainment (7).

## WEB APPENDIX 4: SUPPLEMENTARY TABLES AND FIGURES

**Web Table 1.** Distribution of Self-Reported Human Immunodeficiency Virus Status Among Zambian Adults[a] by Age, Sex and Region, Zambia-South Africa TB and AIDS Reduction Study, 2006-2010.

| Age (years) | Men (N=11484) | | | | | Women (N=22314) | | | | |
|---|---|---|---|---|---|---|---|---|---|---|
| | Reported HIV-negative result | Reported HIV-positive result | Refused to Disclose Test Result | Never Tested | Frequency | Reported HIV-negative result | Reported HIV-positive result | Refused to Disclose Test Result | Never tested | Frequency |
| **Rural, Low Annual Risk of TB Infection (N=9796)** | | | | | | | | | | |
| 18-24 | [b] 38.7 | 0.4 | 2.8 | 58.2 | 1,270 | 61.4 | 2.4 | 3.0 | 33.2 | 2,053 |
| 25-29 | 42.8 | 3.9 | 4.1 | 49.2 | 465 | 66.0 | 8.5 | 3.7 | 21.8 | 1,060 |
| 30-34 | 42.8 | 7.6 | 5.0 | 44.6 | 397 | 54.6 | 13.7 | 4.7 | 26.9 | 780 |
| 35-39 | 44.1 | 10.5 | 4.0 | 41.5 | 354 | 49.0 | 15.4 | 5.1 | 30.4 | 565 |
| 40-49 | 36.9 | 12.5 | 2.6 | 48.0 | 425 | 38.8 | 12.2 | 3.5 | 45.5 | 745 |
| 50+ | 25.4 | 5.2 | 1.5 | 67.8 | 653 | 19.0 | 5.2 | 1.6 | 74.1 | 1,029 |
| All age | 37.6 | 5.0 | 3.1 | 54.4 | 3,564 | 50.5 | 7.7 | 3.4 | 38.5 | 6,232 |
| **Urban, Low Annual Risk of TB Infection (N=7339)** | | | | | | | | | | |
| 18-24 | 41.7 | 0.6 | 2.1 | 55.6 | 981 | 54.6 | 3.4 | 4.7 | 37.3 | 1,655 |
| 25-29 | 44.4 | 5.3 | 5.6 | 44.7 | 360 | 58.5 | 8.8 | 5.6 | 27.0 | 907 |
| 30-34 | 38.5 | 8.6 | 3.3 | 49.7 | 338 | 49.0 | 15.7 | 6.3 | 29.0 | 635 |
| 35-39 | 32.2 | 17.0 | 4.8 | 46.1 | 230 | 36.8 | 20.7 | 6.4 | 36.1 | 421 |
| 40-49 | 29.5 | 13.3 | 4.4 | 52.7 | 315 | 35.4 | 16.6 | 4.6 | 43.4 | 523 |
| 50+ | 27.0 | 6.4 | 1.3 | 65.3 | 392 | 22.3 | 6.5 | 1.7 | 69.4 | 582 |
| All age | 37.2 | 6.1 | 3.1 | 53.6 | 2,616 | 46.9 | 9.5 | 4.9 | 38.7 | 4,723 |
| **Urban (not Lusaka), High Annual Risk of TB Infection (N=6565)** | | | | | | | | | | |
| 18-24 | 43.7 | 0.3 | 1.5 | 54.5 | 890 | 66.2 | 2.6 | 2.1 | 29.1 | 1,478 |
| 25-29 | 49.7 | 3.5 | 2.2 | 44.7 | 318 | 70.0 | 8.5 | 4.1 | 17.5 | 813 |
| 30-34 | 44.6 | 8.9 | 1.9 | 44.6 | 258 | 59.6 | 14.6 | 4.4 | 21.4 | 542 |
| 35-39 | 47.8 | 10.0 | 2.0 | 40.3 | 201 | 51.0 | 16.0 | 4.1 | 28.9 | 363 |
| 40-49 | 35.4 | 11.4 | 4.1 | 49.1 | 271 | 39.7 | 18.7 | 2.7 | 38.9 | 486 |
| 50+ | 30.1 | 3.8 | 1.1 | 64.9 | 365 | 27.6 | 5.7 | 1.4 | 65.3 | 580 |
| All age | 41.9 | 4.4 | 1.9 | 51.8 | 2,303 | 56.5 | 8.7 | 2.9 | 31.9 | 4,262 |
| **Lusaka, High Annual Risk of TB Infection (N=10098)** | | | | | | | | | | |
| 18-24 | 32.8 | 0.3 | 1.4 | 65.5 | 1,153 | 61.7 | 2.6 | 1.9 | 33.8 | 2,618 |
| 25-29 | 40.9 | 2.2 | 2.2 | 54.7 | 508 | 65.2 | 8.8 | 4.0 | 22.0 | 1,370 |
| 30-34 | 34.3 | 6.1 | 2.8 | 56.9 | 362 | 59.7 | 13.3 | 2.0 | 24.9 | 959 |
| 35-39 | 34.3 | 13.1 | 2.2 | 50.4 | 268 | 49.2 | 22.4 | 2.1 | 26.3 | 585 |
| 40-49 | 34.6 | 11.4 | 2.5 | 51.4 | 280 | 41.5 | 16.2 | 1.5 | 40.8 | 779 |
| 50+ | 28.4 | 4.4 | 1.9 | 65.3 | 430 | 23.7 | 6.7 | 0.8 | 68.8 | 786 |
| All age | 34.0 | 4.1 | 1.9 | 59.9 | 3,001 | 54.6 | 8.8 | 2.2 | 34.4 | 7,097 |

Abbreviations: HIV, human immunodeficiency virus; TB, tuberculosis.

[a] Participants responded to a 2010 survey on the prevalence of tuberculosis and human immunodeficiency virus and had an evaluable TB sputum sample

[b] Row percentages

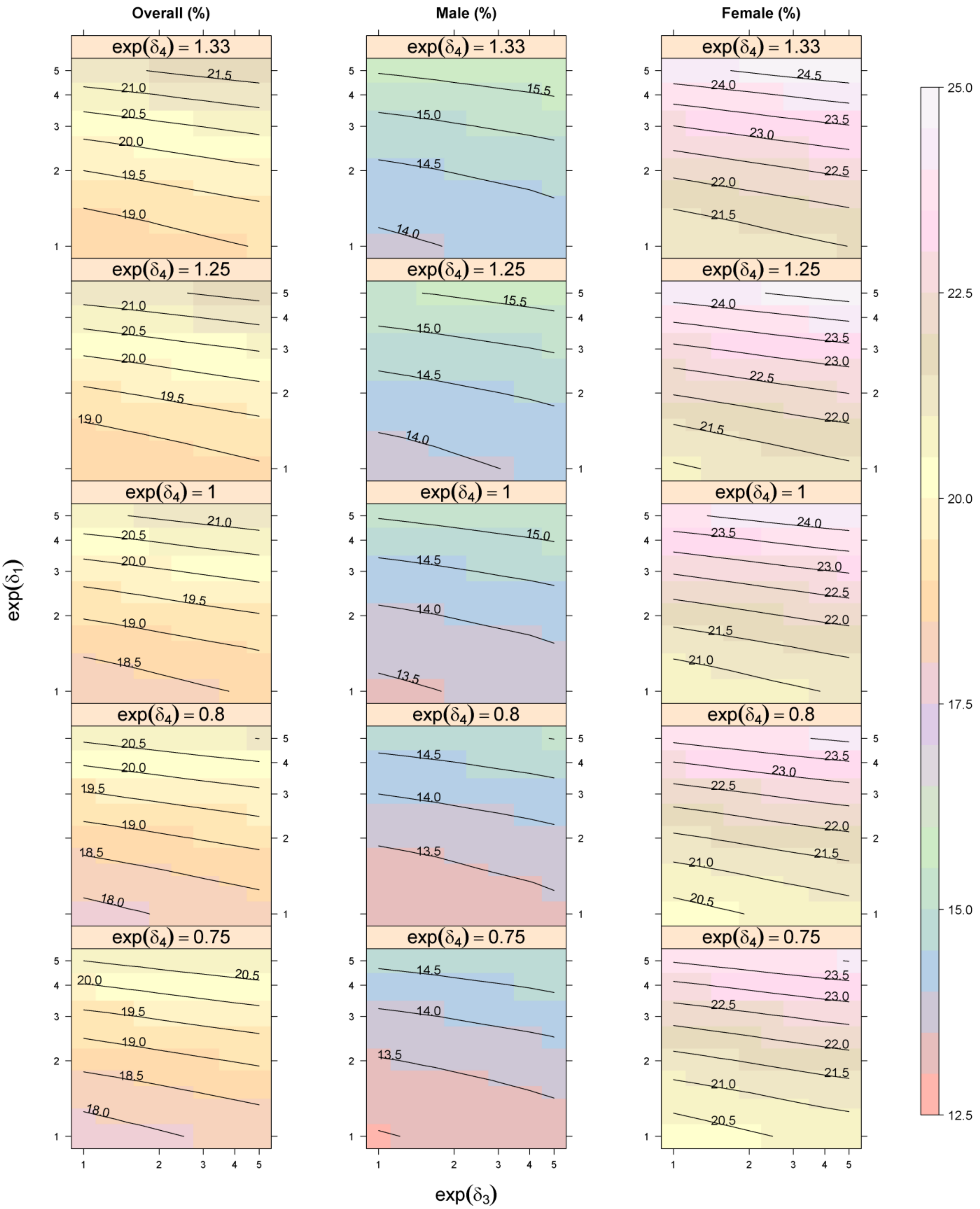


**Web Figure 3.** Filled contour plot of overall and sex-specific human immunodeficiency virus (HIV) prevalence estimates by degree of subgroup-specific departure from the missing at random (MAR) assumption in the HIV test result variable, Zambia-South Africa TB and AIDS Reduction Study, 2006-2010.

$\delta_1$, $\delta_3$ and $\delta_4$ capture the degree of departure from MAR for individuals who self-reported as HIV-negative, individuals who refused to disclose their status and individuals who had never been tested, respectively. $\delta_2$ captures the degree of departure from MAR for individuals who self-reported as HIV-positive and is fixed at zero.

**Web Table 2.** Estimated Odds Ratios from a Logistic Regression of Human Immunodeficiency Virus Test Result Against Educational Attainment, Age, Sex and Region Fitted Among Zambian Adults[a], Zambia-South Africa TB and AIDS Reduction Study, 2006-2010.

| | Complete Case Analysis | | Best Case Analysis[b] | | Worst Case Analysis[c] | | Multiple Imputation under MAR[d] | |
|---|---|---|---|---|---|---|---|---|
| | **OR** | **95% CI** | **OR** | **95% CI** | **OR** | **95% CI** | **OR** | **95% CI** |
| **Educational Attainment** | | | | | | | | |
| None | 0.81 | 0.68, 0.97 | 0.80 | 0.68, 0.95 | 0.98 | 0.88, 1.09 | 0.80 | 0.69, 0.94 |
| Primary | 1.00 | Referent | 1.00 | Referent | 1.00 | Referent | 1.00 | Referent |
| Lower secondary | 1.02 | 0.93, 1.11 | 0.95 | 0.87, 1.03 | 1.09 | 1.03, 1.16 | 1.03 | 0.95, 1.11 |
| Upper secondary | 0.75 | 0.68, 0.83 | 0.67 | 0.61, 0.74 | 1.17 | 1.10, 1.24 | 0.77 | 0.70, 0.85 |
| College/university | 0.64 | 0.55, 0.75 | 0.51 | 0.44, 0.59 | 1.34 | 1.23, 1.46 | 0.66 | 0.57, 0.76 |
| **Sex** | | | | | | | | |
| Male | 1.00 | Referent | 1.00 | Referent | 1.00 | Referent | 1.00 | Referent |
| Female | 4.42 | 3.47, 5.63 | 4.30 | 3.38, 5.47 | 1.21 | 1.12, 1.31 | 4.33 | 3.42, 5.48 |
| **Age, years** | | | | | | | | |
| 18-24 | 1.00 | Referent | 1.00 | Referent | 1.00 | Referent | 1.00 | Referent |
| 25-29 | 5.49 | 4.12, 7.32 | 5.18 | 3.90, 6.89 | 1.40 | 1.25, 1.58 | 5.55 | 4.25, 7.24 |
| 30-34 | 9.94 | 7.53, 13.12 | 8.85 | 6.73, 11.63 | 1.82 | 1.61, 2.07 | 9.79 | 7.51, 12.77 |
| 35-39 | 14.37 | 10.82, 19.07 | 11.67 | 8.85, 15.38 | 2.40 | 2.09, 2.75 | 14.08 | 10.84, 18.30 |
| 40-49 | 14.41 | 10.95, 18.97 | 11.64 | 8.90, 15.22 | 2.36 | 2.07, 2.68 | 14.90 | 11.47, 19.35 |
| 50+ | 4.20 | 3.13, 5.64 | 3.79 | 2.83, 5.07 | 1.40 | 1.25, 1.57 | 4.34 | 3.26, 5.78 |
| **Age:Sex** | | | | | | | | |
| 18-24:Female | 1.00 | Referent | 1.00 | Referent | 1.00 | Referent | 1.00 | Referent |
| 25-29:Female | 0.44 | 0.32, 0.60 | 0.42 | 0.31, 0.58 | 1.14 | 0.99, 1.32 | 0.44 | 0.33, 0.59 |
| 30-34:Female | 0.34 | 0.25, 0.46 | 0.32 | 0.24, 0.43 | 1.14 | 0.98, 1.32 | 0.35 | 0.27, 0.47 |
| 35-39:Female | 0.27 | 0.20, 0.38 | 0.27 | 0.20, 0.37 | 0.96 | 0.81, 1.14 | 0.30 | 0.23, 0.40 |
| 40-49:Female | 0.21 | 0.15, 0.28 | 0.22 | 0.17, 0.30 | 0.77 | 0.66, 0.90 | 0.21 | 0.17, 0.29 |
| 50+:Female | 0.24 | 0.17, 0.34 | 0.25 | 0.18, 0.34 | 0.84 | 0.73, 0.97 | 0.24 | 0.19, 0.34 |
| **Region and TB risk** | | | | | | | | |
| Rural, low ARTI | 1.00 | Referent | 1.00 | Referent | 1.00 | Referent | 1.00 | Referent |
| Urban, low ARTI | 2.07 | 1.86, 2.30 | 1.85 | 1.68, 2.05 | 1.43 | 1.34, 1.52 | 1.83 | 1.67, 2.01 |
| Urban (not Lusaka), high ARTI | 1.56 | 1.40, 1.74 | 1.54 | 1.38, 1.71 | 1.14 | 1.07, 1.22 | 1.42 | 1.28, 1.57 |
| Lusaka, high ARTI | 1.42 | 1.29, 1.57 | 1.66 | 1.51, 1.82 | 0.76 | 0.72, 0.81 | 1.29 | 1.18, 1.40 |

Abbreviations: ARTI: annual risk of tuberculosis infection; CI, confidence interval; HIV, human immunodeficiency virus; MAR, missing at random; OR, odds ratio; TB, tuberculosis.

[a] Participants responded to a 2010 survey on the prevalence of tuberculosis and human immunodeficiency virus, had an evaluable TB sputum sample and agreed to be tested for HIV (n=23093)

[b] All missing HIV test result values were imputed as positive

[c] All missing HIV test result values were imputed as negative

[d] Imputation model included age, region, active pulmonary TB, household wealth index, educational attainment, current TB treatment, past TB treatment, marital status, diabetes status, smoking status, alcohol consumption, hunger in past 3 months, household crowding, circumcision status (males only), current cough, persistent cough for more than 2 weeks, current chest pain, current fever, current night sweats, current shortness of breath, unintentional weight loss in past month, and self-reported HIV status

**Web Table 3.** Estimated Odds Ratios from a Logistic Regression of Active Pulmonary Tuberculosis Against Human Immunodeficiency Virus Test Result, Educational Attainment, Age, Sex and Region Fitted Among Zambian Adults[a], Zambia-South Africa TB and AIDS Reduction Study, 2006-2010.

| | Complete Case Analysis | | Best Case Analysis[b] | | Worst Case Analysis[c] | | Multiple Imputation under MAR[d] | |
|---|---|---|---|---|---|---|---|---|
| | **OR** | **95% CI** | **OR** | **95% CI** | **OR** | **95% CI** | **OR** | **95% CI** |
| **Education** | | | | | | | | |
| None | 1.88 | 0.97, 3.64 | 1.43 | 0.77, 2.66 | 1.40 | 0.75, 2.59 | 1.45 | 0.78, 2.70 |
| Primary | 1.00 | Referent | 1.00 | Referent | 1.00 | Referent | 1.00 | Referent |
| Lower secondary | 0.71 | 0.45, 1.11 | 0.78 | 0.54, 1.12 | 0.76 | 0.52, 1.09 | 0.77 | 0.54, 1.12 |
| Upper secondary | 0.69 | 0.41, 1.08 | 0.71 | 0.48, 1.04 | 0.65 | 0.44, 0.95 | 0.73 | 0.50, 1.07 |
| College/university | 0.33 | 0.12, 0.93 | 0.29 | 0.13, 0.64 | 0.24 | 0.11, 0.53 | 0.30 | 0.14, 0.66 |
| **Sex** | | | | | | | | |
| Male | 1.00 | Referent | 1.00 | Referent | 1.00 | Referent | 1.00 | Referent |
| Female | 0.89 | 0.42, 1.88 | 0.98 | 0.52, 1.86 | 1.04 | 0.55, 1.97 | 0.85 | 0.45, 1.63 |
| **Age, years** | | | | | | | | |
| 18-24 | 1.00 | Referent | 1.00 | Referent | 1.00 | Referent | 1.00 | Referent |
| 25-29 | 1.72 | 0.69, 4.31 | 3.28 | 1.64, 6.55 | 3.47 | 1.74, 6.92 | 2.72 | 1.35, 5.48 |
| 30-34 | 3.83 | 1.73, 8.46 | 4.45 | 2.28, 8.69 | 4.86 | 2.50, 9.45 | 3.40 | 1.72, 6.72 |
| 35-39 | 1.35 | 0.47,3.83 | 2.08 | 0.91, 4.78 | 2.28 | 1.00, 5.21 | 1.47 | 0.63, 3.43 |
| 40-49 | 0.99 | 0.33, 2.98 | 2.13 | 0.97, 4.68 | 2.32 | 1.06, 5.09 | 1.50 | 0.67, 3.34 |
| 50+ | 1.39 | 0.54, 3.60 | 1.54 | 0.69, 3.45 | 1.55 | 0.70, 3.47 | 1.35 | 0.60, 3.03 |
| **Age:Sex** | | | | | | | | |
| 18-24:Female | 1.00 | Referent | 1.00 | Referent | 1.00 | Referent | 1.00 | Referent |
| 25-29:Female | 0.61 | 0.21,1.84 | 0.69 | 0.16, 0.92 | 0.38 | 0.16, 0.90 | 0.40 | 0.17, 0.96 |
| 30-34:Female | 0.18 | 0.06, 0.54 | 0.18 | 0.07,0.46 | 0.17 | 0.07, 0.44 | 0.19 | 0.08, 0.50 |
| 35-39:Female | 0.76 | 0.22, 2.78 | 0.59 | 0.21,1.68 | 0.57 | 0.20, 1.62 | 0.65 | 0.23, 1.85 |
| 40-49:Female | 0.67 | 0.17, 2.58 | 0.52 | 0.19, 1.42 | 0.50 | 0.19, 1.36 | 0.62 | 0.23, 1.67 |
| 50+:Female | 0.12 | 0.02, 0.69 | 0.13 | 0.03, 0.56 | 0.13 | 0.03, 0.53 | 0.15 | 0.04, 0.64 |
| **Region and TB risk** | | | | | | | | |
| Rural, low ARTI | 1.00 | Referent | 1.00 | Referent | 1.00 | Referent | 1.00 | Referent |
| Urban, low ARTI | 1.64 | 0.93, 2.87 | 1.69 | 1.10, 2.59 | 1.72 | 1.13, 2.64 | 1.55 | 1.01, 2.38 |
| Urban (not Lusaka), high ARTI | 1.10 | 0.58, 2.09 | 1.32 | 0.83, 2.11 | 1.36 | 1.23, 2.17 | 1.28 | 0.80, 2.04 |
| Lusaka, high ARTI | 2.05 | 1.25, 3.36 | 1.61 | 1.08, 2.41 | 1.83 | 1.75, 2.73 | 1.64 | 1.10, 2.44 |
| **HIV test result** | | | | | | | | |
| Negative | 1.00 | Referent | 1.00 | Referent | 1.00 | Referent | 1.00 | Referent |
| Positive | 4.06 | 2.80, 5.89 | 3.02 | 2.19, 4.17 | 2.37 | 1.75, 3.20 | 4.69 | 3.32, 6.63 |

Abbreviations: ARTI: annual risk of tuberculosis infection; CI, confidence interval; HIV, human immunodeficiency virus; MAR, missing at random; OR, odds ratio; TB, tuberculosis.

[a] Participants responded to a 2010 survey on the prevalence of tuberculosis and human immunodeficiency virus, had an evaluable TB sputum sample and agreed to be tested for HIV (n=23093)

[b] All missing HIV test result values were imputed as positive

[c] All missing HIV test result values were imputed as negative

[d] Imputation model included age, region, active pulmonary TB, household wealth index, educational attainment, current TB treatment, past TB treatment, marital status, diabetes status, smoking status, alcohol consumption, hunger in past 3 months, household crowding, circumcision status (males only), current cough, persistent cough for more than 2 weeks, current chest pain, current fever, current night sweats, current shortness of breath, unintentional weight loss in past month, and self-reported HIV status

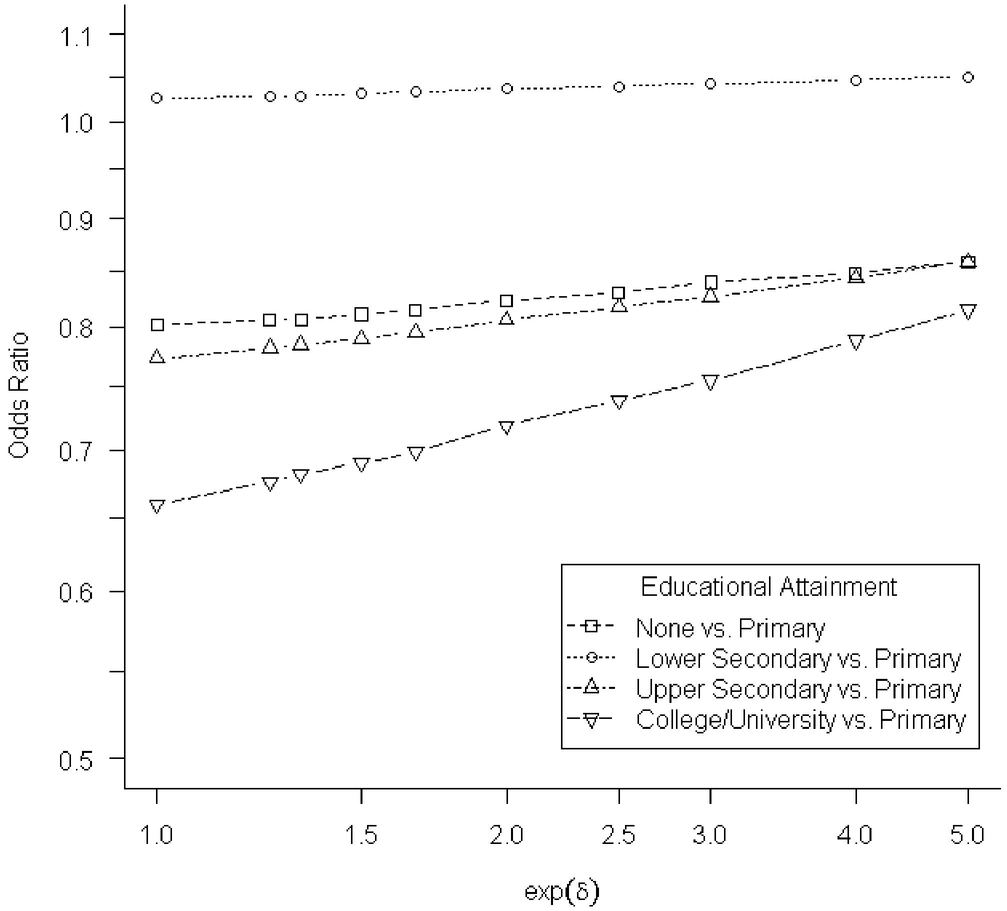


**Web Figure 4.** Association between positive human immunodeficiency virus (HIV) test result and individual educational attainment adjusted for age, sex and region by degree of departure ($\boldsymbol{\delta=\delta_1=\delta_2=\delta_3=\delta_4}$) from the missing at random assumption in the HIV test result variable, Zambia-South Africa TB and AIDS Reduction Study, 2006–2010.

## REFERENCES


1. Alyes H, Muyoyeta M, Du Toit E, et al. Effect of household and community interventions on the burden of tuberculosis in Southern Africa: the ZAMSTAR community-randomised trial. *Lancet.* 2013; **382**(9899):1183-1194.
2. Reniers G, Eaton J. Refusal bias in HIV prevalence estimates from nationally representative seroprevalence surveys. *AIDS.* 2009: **23**(5):621-629.
3. Floyd S, Molesworth A, Dube A, et al. Underestimation of HIV prevalence in surveys when some people already know their status, and ways to reduce the bias. *AIDS.* 2013; **27**(2):223-242.
4. van Buuren S, Groothuis-Oudshoorn K. MICE: Multivariate imputation by chained equations in R. *J Stat Softw.* 2011; **45**(3):1-67.
5. Valeri L, VanderWeele TJ. Mediation analysis allowing for exposure-mediator interactions and causal interpretation: Theoretical assumptions and implementation with SAS and SPSS macros. *Psychol Methods.* 2013; **18**(2):137-150.
6. VanderWeele TJ, Robinson WR. On the causal interpretation of race in regressions adjusting for confounding and mediating variables. Epidemiology. 2014; **24**:473-84.
7. Naimi AI, Kaufman JS. Counterfactual Theory in Social Epidemiology: Reconciling Analysis and Action for the Social Determinants of Health. *Curr Epidemiol Rep*. 2015; **2**(1):52-60.
